# Circulating tumor cells (CTCs) enumeration and machine-learning based diagnostic biomarkers for breast cancer detection

**DOI:** 10.1186/s12885-026-15741-9

**Published:** 2026-03-03

**Authors:** Chun-Yu Liu, Yu-Hsiang Lin, Yi-Fang Tsai, Po-Yen Lu, Ji-Lin Chen, Yu-Hsuan Li, Chi-Cheng Huang, Yen-Shu Lin, Ta-Chung Chao, Chin-Jung Feng, Chih-Yi Hsu, Jen-Hwey Chiu, Chyong-Mei Chen, Ling-Ming Tseng

**Affiliations:** 1 School of Medicine, College of Medicine, National Yang Ming Chiao Tung University, Taipei, 11217 Taiwan; 2 Comprehensive Breast Health Center, Department of Surgery, Taipei Veterans General Hospital, No. 201, Section 2, Shipai Road, Taipei, 11217 Taiwan; 3 Division of Medical Oncology, Department of Oncology, Taipei Veterans General Hospital, Taipei, 11217 Taiwan; 4 Division of Breast Surgery, Department of Surgery, Taipei Veterans General Hospital, Taipei, 11217 Taiwan; 5 Institute of Public Health, College of Medicine, National Yang Ming Chiao Tung University, No. 155, Sec. 2, Linong St., Beitou Dist, Taipei City, 112 Taiwan; 6 Department of Public Health, College of Public Health, National Taiwan University, Taipei, Taiwan; 7 Department of Pathology and Laboratory Medicine, Taipei Veterans General Hospital, Taipei, 11217 Taiwan; 8 Institute of Traditional Medicine, School of Medicine, National Yang Ming Chiao Tung University, Taipei, 11217 Taiwan

**Keywords:** Circulating tumor cells, Machine-learning algorithms, Breast cancer, Monte carlo cross-validation, Ensemble classifier

## Abstract

**Background:**

Circulating tumor cells (CTCs) are detectable in early-stage cancer and may enable early cancer detection. We evaluated a CTC-based assay as a complementary biomarker for breast cancer detection in an Asian population with a high prevalence of dense breast tissue.

**Methods:**

In this single-center, prospective, blinded study, peripheral blood from Taiwanese women with breast cancer and healthy controls was analyzed using a CTC-enumeration platform (CMx) based on biomarker expression (cytokeratin 18 [CK18], mammaglobin [MGB], CD45), cell morphometry, and nuclear features. A machine-learning model integrating CTC biomarkers with age, white blood cell (WBC) count, and platelet count was developed to assess classification performance, providing proof-of-concept for combining CTC-derived and routine blood parameters in breast cancer risk assessment.

**Results:**

A total of 228 breast cancer patients and 170 healthy controls were included. Age and CK18- and MGB-positive CTC counts differed significantly between groups, whereas WBC and platelet counts did not. An ensemble linear support vector machines model incorporating age and CTC features achieved an area under the curve of 0.85 (95% CI, 0.73–0.96) in the independent test cohort, with high sensitivity (0.93), positive predictive value (0.74), and negative predictive value (0.86), but modest specificity (0.57). In the exploratory BI-RADS 3/4 subgroup, the model identified all cancer cases (sensitivity 1.00), with a specificity of 0.44 and overall accuracy of 0.79.

**Conclusions:**

This study demonstrates the feasibility of combining CTC enumeration with machine learning for breast cancer detection and supports the need for future large-scale, multicenter, multiethnic prospective external validation.

**Supplementary Information:**

The online version contains supplementary material available at 10.1186/s12885-026-15741-9.

## Background

 Breast cancer is the most frequently diagnosed cancer globally and is the leading cause of cancer mortality among females in many countries [1]. Early detection of breast cancer significantly increases survival, improves patient outcomes, and reduces disease burden. Currently, breast cancer screening mostly depends on mammography, which has been proven to be associated with reduced breast cancer mortality [2, 3]. However, the risks of overdiagnosis of indolent disease and false-positive results related to mammography screening remain a challenge. Thus, there is an increasing need to develop complementary diagnostic tools for breast cancer that are less invasive but capable of providing additional clinical guidance. While serum markers such as CEA and CA 15 − 3 are utilized in routine physical exams as potential indicators, none are sufficiently sensitive and they are more commonly used to monitor responses to cancer treatment and disease recurrence. Hence, alternative methods for tumor liquid biopsy to support early cancer detection are gaining momentum.

Liquid biopsy has emerged as a transformative approach in cancer detection, offering a less invasive alternative to conventional tissue biopsy, which remains the diagnostic gold standard but is limited by invasiveness, challenges in repeated sampling, and vulnerability to tumor heterogeneity. This strategy encompasses diverse analytes, including circulating tumor cells (CTCs), circulating tumor DNA (ctDNA), exosomes, microRNAs, circulating RNAs, tumor-educated platelets, and autoantibodies, each with unique strengths and limitations [4]. CTCs, though historically recognized as valuable indicators of tumor progression, are extremely rare in peripheral blood and often missed by EpCAM-dependent detection methods [5]. ctDNA, in contrast, provides real-time insights into tumor genetics through mutation profiling, methylation analysis, and fragmentomics, but its low abundance in early-stage disease remains a major barrier. Exosomes and circulating RNAs offer stable carriers of nucleic acids and proteins reflective of the tumor microenvironment, although standardization of isolation methods is still lacking. Conventional serum proteins such as CEA and CA 15 − 3 suffer from low sensitivity and specificity, but efforts to combine multiple proteins or employ high-throughput proteomics are ongoing [6]. Despite these advances, most liquid biopsy approaches remain limited by insufficient sensitivity in early-stage cancers, difficulties in pinpointing the tissue of origin, and performance declines when applied to asymptomatic populations. In particular, CTCs offer a distinct advantage by preserving intact cellular architecture, which enables simultaneous morphological, genomic, and transcriptomic analyses from a single source. Unlike ctDNA or other acellular biomarker-based approaches, CTC assays allow the study of viable tumor cells, supporting downstream functional assays and phenotypic profiling that multi-omics strategies cannot capture [7, 8]. Although multi-marker liquid biopsy panels can improve detection sensitivity, their analytical complexity, cost, and standardization challenges may limit broad clinical adoption. A CTC-focused strategy therefore represents a simpler yet complementary approach for breast cancer risk assessment, particularly valuable in populations with dense breast tissue where conventional imaging demonstrates reduced sensitivity [9]. Furthermore, integrating CTC analysis with artificial intelligence (AI) and machine-learning (ML) may enhance diagnostic resolution, mitigate tumor heterogeneity, and improve both sensitivity and specificity for early cancer detection.

CTCs represent tumor cells that detach from the primary tumor site and enter the bloodstream via intravasation, thereby contributing to the metastatic dissemination process within the peripheral blood circulation system [10]. Previous investigations focusing on micrometastasis within the bone marrow of breast cancer patients elucidated early dissemination to distant sites, even among individuals with small, early-stage tumors [11]. Hence, CTCs present in the bloodstream could serve as initial indicators of the early phases of tumor metastasis, suggesting their potential as prognostic indicators or even as early diagnostic markers for breast cancer.

Clinically, outcome prediction may be more practical than estimating the effects of risk or diagnostic factors, as it can positively impact clinical decision-making. From this perspective, ML classifiers have been successfully used to predict diseases [12–18]. Furthermore, ML classifiers are known for their flexibility and robustness in avoiding the strong assumptions typically underlying standard statistical models, particularly concerning the assumed relationships between outcomes and features/covariates [12, 14, 16–18]. In this study, we aim to utilize CTCs in conjunction with ML to create a diagnostic tool for detecting breast cancer.

## Methods

### Patients and specimens

**Fig. 1:**
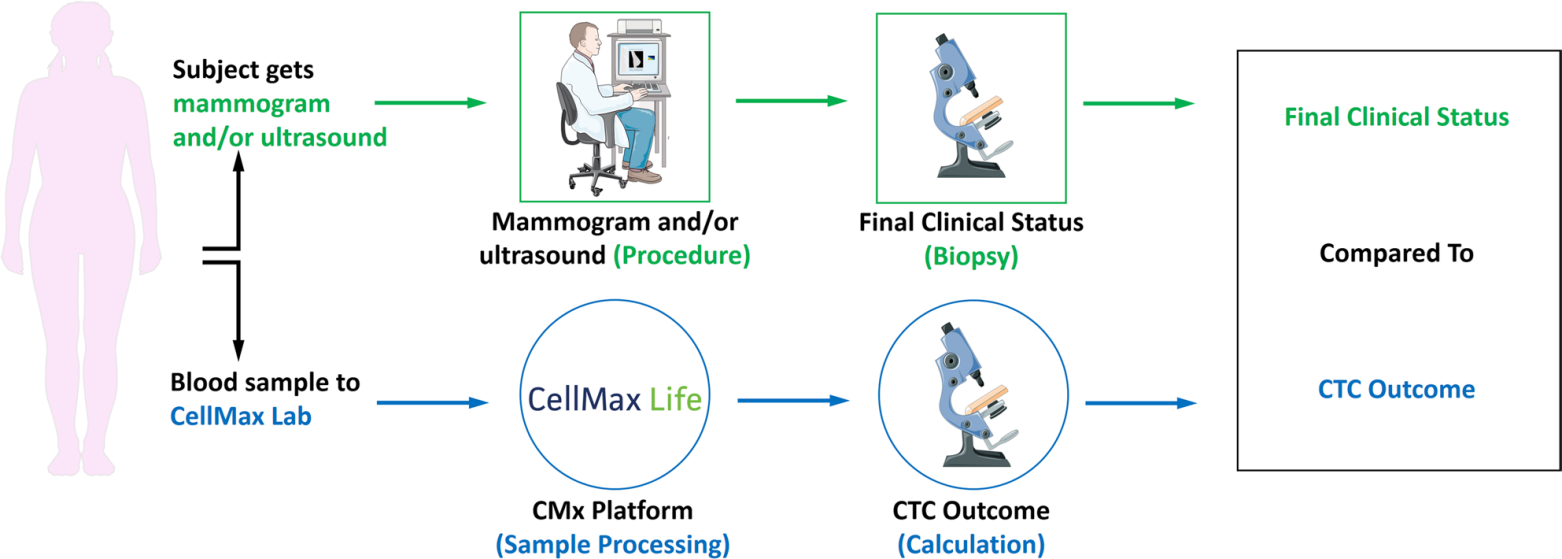
Study design for evaluating the performance of the CMx test. Peripheral blood was collected prior to diagnostic confirmation and, when applicable, at the same visit as breast imaging (mammography or ultrasound). Blood samples were processed using the CMx platform to enumerate CTCs. Imaging findings and histopathology (biopsy) were used to establish the final clinical status. CTC outcomes derived from the CMx test were subsequently compared with the final clinical diagnosis to evaluate test performance

This study was approved by the Institutional Review Board of Taipei Veterans General Hospital (TPEVGH IRB No. 2017-10-016AC). Written informed consent was obtained from all participants prior to peripheral blood sampling and data collection, which were conducted in compliance with the Helsinki Declaration. For most participants, blood collection was performed on the same day as mammography or ultrasound. In cases where the procedures did not coincide, the interval between imaging and blood draw was recorded, with a median interval of 14 days. Eligible participants were adult women with pathology-confirmed breast cancer (including stage 0 ductal carcinoma in situ) and healthy women with either benign breast lesions (e.g., fibroadenoma or fibrocystic change) or normal breast findings on imaging [Breast Imaging Reporting and Data System (BI-RADS) category 1 by breast sonography and/or mammography]. Patients with lobular carcinoma in situ (LCIS) were excluded, as LCIS is generally regarded as a non-invasive, risk-associated lesion rather than a malignant endpoint, and most clinical trials and risk prediction models do not classify LCIS as breast cancer per se [19]. Furthermore, CTC characteristics in LCIS remain undefined in published literature, making biological interpretation challenging and potentially confounding if such cases were included. Between February 2018 and November 2021, a total of 398 patients were enrolled, comprising 228 individuals with breast cancer and 170 individuals categorized as benign or healthy. All participants were followed for a minimum of two years. The study was designed to evaluate the performance of the CMx platform for CTC detection and enumeration in breast cancer (Fig. 1).

### CTCs detection and enumeration (CMx platform)

**Fig. 2:**
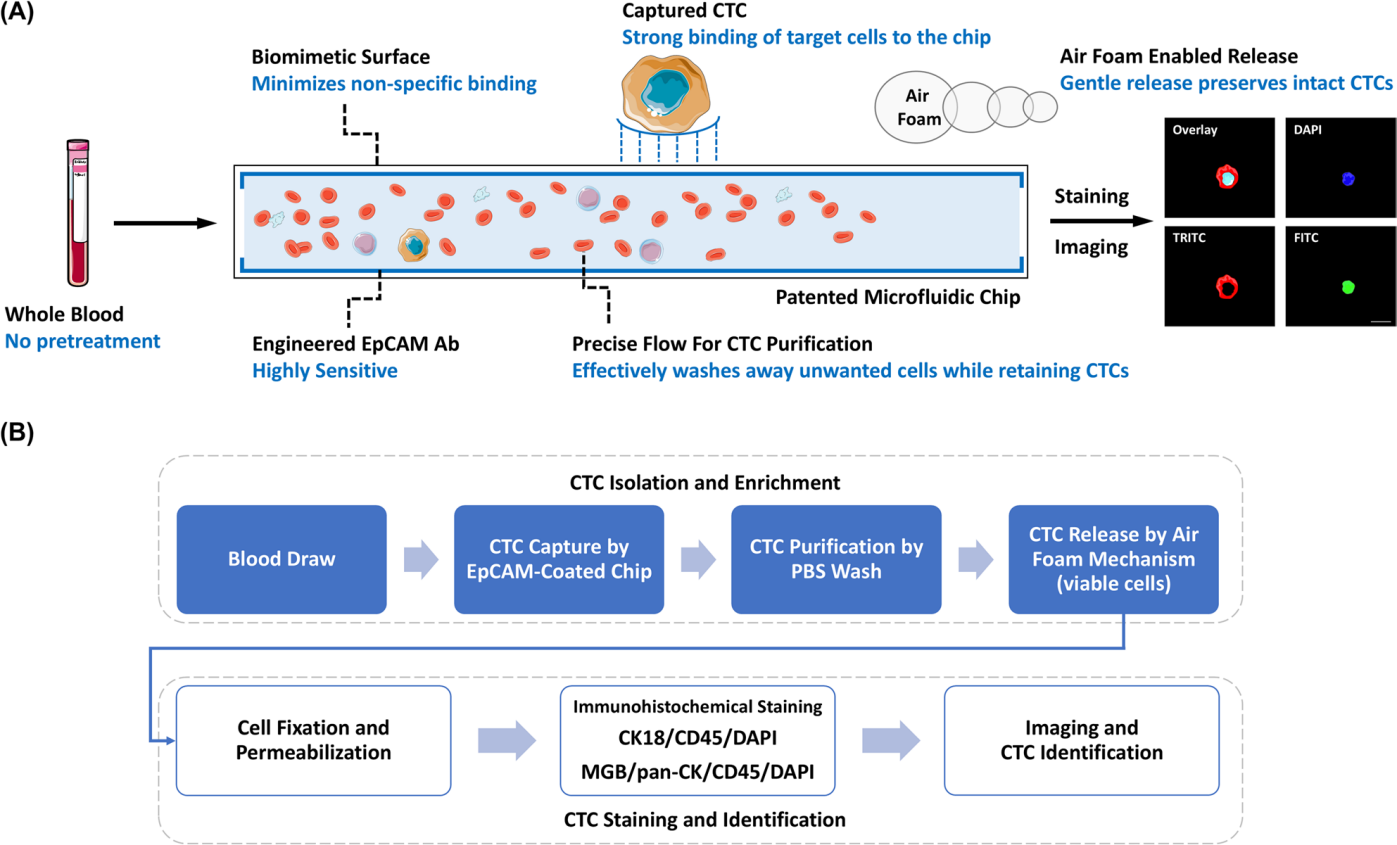
Schematic illustration of the microfluidic platform for detecting CTCs. **A** The CMx chip consists of a microfluidic channel coated with high-affinity anti-EpCAM antibodies on a supported lipid bilayer and a biomimetic, non-fouling surface to minimize non-specific binding. Whole blood is introduced directly into the chip without pretreatment at a controlled flow rate, allowing efficient capture of EpCAM-expressing CTCs. Captured cells can subsequently be released by an air-foam mechanism for downstream molecular analyses. **B** Workflow of CTC identification: after peripheral blood collection, CTCs are captured on the EpCAM-coated CMx chip, washed with phosphate-buffered saline (PBS) to remove non-target cells, and either released in viable form using the air-foam method or fixed and permeabilized for immunofluorescence staining. CTCs are identified as DAPI-positive with CK18-positive cytoplasm or MGB-positive membrane and negative for the leukocyte marker CD45

The analytical validation of the CMx platform for rare CTC detection has been previously reported (Fig. 2A) [20–22]. Peripheral blood samples from donors were first collected in Vacutainer tubes containing EDTA anticoagulant (BD Biosciences, USA) and a cell preservative (Streck, USA). Subsequently, 2 mL of blood was introduced into a microfluidic EpCAM-coated CMx chip, operated at a controlled flow rate of 1.5 mL/h to capture CTCs. Following infusion, loosely bound cells were removed with phosphate-buffered saline, and the captured cells were released using air foams that gently detach the supported lipid bilayer from the chip surface, thereby liberating intact cells without harsh disruption of antigen–antibody bonds [20]. EpCAM, a homophilic type I transmembrane glycoprotein, has been widely adopted as a cell surface marker of epithelial carcinomas [23]. Mammaglobin (MGB), a 93-amino-acid glycoprotein, has been implicated as a diagnostic marker for breast carcinoma [24]. Cytokeratin 18 (CK18), an intermediate filament protein of the acidic type I cytokeratin family, is consistently expressed in many epithelial cancers, particularly adenocarcinomas [25], and has been applied in CTC detection [26, 27]. Prior studies have also explored the use of MGB as a breast cancer–specific CTC marker, in combination with EpCAM, CD45, and pan-CK for immunophenotyping [28]. In this study, MGB was used as an exploratory feature in combination with CK18 and patient age within an ensemble ML model to improve discrimination between breast cancer patients and healthy controls.

### CTC staining and identification

In this study, released cells were stained with antibodies against CK18, MGB, pan-CK (guinea pig anti-Cytokeratin 8/18; OriGene, catalog #BP5007, USA), and CD45, together with the nuclear stain 4′,6-diamidino-2-phenylindole (DAPI). CTCs were defined and enumerated through a standardized three-step workflow. First, each patient sample was divided onto two slides, with cells mounted on a 10-mm membrane and imaged across four fluorescence channels using a Leica autofocus system. The first slide was stained with TRITC-conjugated anti-CK18, FITC-conjugated anti-CD45, and DAPI; the second slide was stained with Cy5-conjugated anti–pan-CK, TRITC-conjugated anti-MGB, FITC-conjugated anti-CD45, and DAPI. For each slide, 100 frames were automatically captured and stitched to generate composite image volumes for each channel. Second, the stitched images were analyzed using custom AI software that identified candidate cell-like regions. Each candidate was assigned a confidence index, trained iteratively against confirmed CTCs and white blood cells (WBCs), and flagged for morphology review. Finally, candidate events were examined in the CellReviewer platform according to strict morphological and immunophenotypic criteria: round-to-oval cell shape, diameter of 8–40 μm, positive cytoplasmic/membrane CK18 or MGB signal, CD45 negativity, and a DAPI-positive nucleus (Fig. 2B). WBCs were excluded based on their multilobed nuclear morphology. Enumeration was performed by a trained technician and verified by an expert pathologist when required.

### Machine-learning models establishment and statistics

**Fig. 3:**
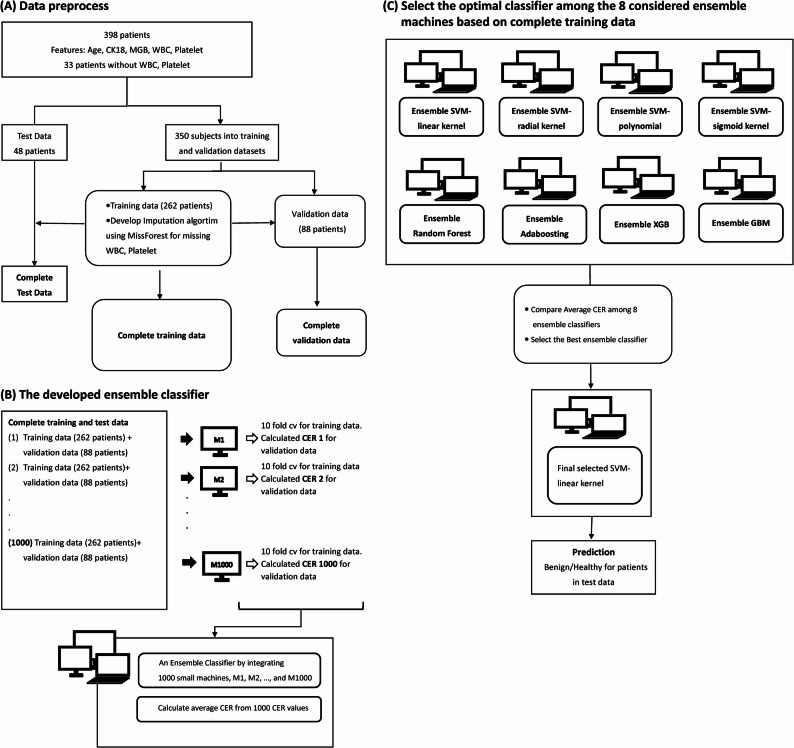
Research framework diagram. **A** Data preprocess for training, validation and test datasets. The imputation for missing data is implemented by the MissForest algorithm using the training data. **B** The proposed ensemble machine algorithm based on MCCV procedure, in which the random data-splitting and model-building steps are repeated up to 1,000 times. The notation M1, M2, …, M1000, *…* represents a continuing sequence of models that follow a regular pattern. The ellipsis (“…”) indicates that intermediate elements are omitted for brevity but are implied to exist in the same format (e.g., M3, M4, …, M999). After imputing missing data as described in (A), machine is developed by grid search method and 10-fold cv for the training data and then calculate CER for validation data. Model building procedure is repeated up to 1,000 times. (C) The best machine chosen from 8 algorithms are based on the complete training data and predictions on the test data through a majority voting system from the 1000 machines of the best predictive model

To predict outcomes, the analysis relied on a set of features: age, CK18, MGB, WBC, and platelet (referred to as Model 1). Our study aimed to develop a predictive model for classifying individuals into two categories: healthy/benign or diagnosed with cancer. Various ML algorithms were considered, including support vector machines (SVM) with different kernels, gradient boosting machine (GBM), random forest (RF), adaptive boosting (Adaboosting), and extreme gradient boosting (XGB) [16, 17]. Due to the limited sample size, a novel Monte Carlo cross-validation (MCCV) procedure was devised on the training set to construct an ensemble classifier [29]. To ensure robustness, 48 subjects were randomly selected for a test dataset using a stratified random sampling method to maintain proportions similar to the original data between healthy and cancer subjects (Fig. 3A). The MCCV procedure involved randomly splitting the remaining 350 subjects into training and validation datasets 1000 times, with 75% (*n* = 262) allocated to training and 25% (*n* = 88) to validation in each split. Within each split, ML models were trained on the training data using 10-fold cross-validation. Hyperparameters were tuned within prespecified ranges appropriate for each algorithm (Table S1). For example, SVM models varied the cost parameter (0.01–1000); GBM models tuned interaction depth (1–9), shrinkage (0.01, 0.1, 0.2), n.minobsinnode (5–20), bag.fraction (0.5–1.0), and n.trees (10,000); XGBoost (XGB) models tuned eta (0.01, 0.1, 0.2), max_depth (1–9), min_child_weight (1–9), subsample (0.7–1.0), and colsample_bytree (0.7–1.0). The optimal hyperparameters were selected by minimizing the classification error rate (CER) or maximizing accuracy in the validation folds. This process was repeated across 1000 random splits to generate an ensemble of “small” models for each algorithm, which were subsequently aggregated to produce the final ensemble classifier (Fig. 3B). This process was also repeated to integrate 1000 trained “small” ML models into an ensemble classifier for each of the eight ML algorithms considered. The performance of each trained “small” ML model was evaluated by CER for the corresponding validation set. The ensemble classifier with the lowest average estimated CER of validation sets, obtained from the 1000 optimized ML models across repeated splits, was selected as the proposed model (Fig. 3C). The proposed approach differs from traditional MCCV, as it integrates all “small” models from each splitting step into the ensemble classifier, making it particularly suitable for datasets with limited samples. Notably, within the total cohort, 33 individuals identified as healthy had missing data for WBC and platelet counts. Given the missing data for some patients’ WBC and platelet counts, imputation was performed using the missForest R package within each split before implementing the ML algorithms as our data preprocessing method (Fig. 3A). The same MCCV procedure was also applied to features excluding WBC and platelet (referred to as Model 2) for all eight ML algorithms, aiming to assess the impact of imputation and the contribution of these specific features (Fig. 3C). Ultimately, the best predictive model, determined by the smallest average CER derived from 1000 optimal ML models across repeated splitting, was employed for predictions on the test data through a majority voting system from the 1000 machines of the best predictive model. After obtaining the predicted probabilities, a patient was classified as having breast cancer if the probability exceeded a pre-defined threshold of 0.5. This threshold was selected a priori for this proof-of-concept evaluation, as it represents the theoretically grounded decision point for a Bayes classifier when class priors are approximately balanced and misclassification costs are assumed to be equal [30].

The primary analyses, including the construction and validation of Models 1 and 2, were pre-specified as part of the study design. Subsequent subgroup analyses by cancer stage, molecular subtype, breast density, and BI-RADS category were exploratory and not pre-specified. To further characterize the performance trade-offs across different operating points, a sensitivity analysis for the test data was conducted by evaluating model metrics across a range of probability thresholds (0.3–0.8). This analysis was intended to illustrate threshold-dependent performance variations rather than to define a post-hoc optimized cutoff. All analyses were conducted using R software (version 4.2.1), with statistical significance set at a two-sided *p*-value of < 0.05. The source code for this model is available at https://github.com/CYL-lab/SVM_linear_Model (v1.0.0, commit 9147380). To ensure long-term reproducibility, a copy of the source code including environment specifications (sessionInfo) is also provided as Supplementary Material.

## Results

### Baseline clinical characteristics

The baseline clinical and biological characteristics for the entire cohort are summarized (Table 1). To ensure the integrity of model development, these characteristics are further detailed separately for the training dataset (*n* = 350; Table S2) and the testing dataset (*n* = 48; Table S3). Comparison between the two cohorts revealed no statistically significant differences in age or any biomarker levels (all *p* > 0.05; Table S4). All features were continuous, and the median as well as the first and third quartiles were reported, due to the departure from normality observed in the Shapiro test results. The frequency distribution of cases was presented (Table 2). Furthermore, the distribution of CTC enumeration data, focusing on CK18 and MGB expression, was displayed (Figure S1).

**Table 1:** Demographic characteristics of all patients

Characteristics	All patients (*n* = 398)	Cancer (*n* = 228)	Benign/Healthy (*n* = 170)	*p*-value
Age (years)	51 (43, 62)	56 (46.75, 64)	46.5 (34, 54)	< 0.001*
CK18	3 (1, 6)	3 (1, 7)	2 (1, 4)	< 0.001*
MGB	4 (2, 8)	5 (2, 10)	3 (1.25, 7)	< 0.001*
WBC (/uL)	6300 (5400, 7700)	6400 (5400, 7700)	6300 (5400, 7700)^§^	0.688
Platelet (/uL)	255,000 (217000, 293000)	254,000 (216000, 291000)	255,000 (224000, 297000)^§^	0.527

*CK18* cytokeratin 18, *MGB* mammaglobin, *WBC* white blood cell

^§^Summarized by deleting the missing values (*n* = 33 in Benign/Healthy group)

Median (Q1, Q3)

Mann–Whitney–Wilcoxon test. *p-values < 0.05 were considered statistically significant for comparisons between cancer and benign/healthy groups

**Table 2:** Distribution of cases

Characteristic	Cancer (%) *n* = 228	Benign/Healthy (%) *n* = 170
Cancer stage		
0	16 (7.0)	–
1	71 (31.1)	–
2	99 (43.4)	–
3	26 (11.4)	–
4	15 (6.6)	–
NA	1 (0.4)	–
Subtypes		
ER+HER2−	144 (63.2)	–
ER+HER2+	26 (11.4)	–
ER−HER2+	22 (9.6)	–
ER−HER2−	36 (15.8)	–
Breast density		
Category B	11 (4.8)	5 (2.9)
Category C	189 (82.9)	96 (56.5)
Category D	19 (8.3)	12 (7.1)
NA	9 (3.9)	57 (33.5)
BI-RADS category		
0	2 (0.9)	0 (0.0)
1	0 (0.0)	15 (8.8)
2	2 (0.9)	63 (37.1)
3	2 (0.9)	16 (9.4)
4	122 (53.5)	75 (44.1)
5	30 (13.2)	0 (0.0)
6	67 (29.4)	0 (0.0)
NA	3 (1.3)	1 (0.6)

Category B: scattered areas of fibroglandular density, Category C: heterogeneously dense. Category D: extremely dense

*NA* not available, *ER* estrogen receptor, *HER2* human epidermal growth factor receptor 2

### Model performance

Performances of the ensemble machine, evaluated from its 1000 small ML models for the corresponding validation sets, were summarized using the average area under the curve (AUC) and the average CER. AUC and CER for all eight models are reported in Table 3. Among these models, the ensemble classifiers based on SVM with linear and radial kernels, along with GBM models, demonstrated comparable performances under the same set of features. Notably, these models outperformed the remaining five models in terms of either CER or AUC. Considering computational efficiency, linear and radial SVMs were preferred over GBM, which required substantially greater memory and computation time (≈ 11X slower than SVM-linear; SVM-radial ≈ 1.3X slower). Consequently, the GBM-based ensemble was excluded. Although SVM-linear and SVM-radial showed comparable performance in the primary analysis (Table 3), Supplementary experiments (additional comparisons using datasets split with different random seeds, data not shown) revealed superior stability of the linear SVM, whereas the radial SVM produced inconsistent or inferior results. Therefore, SVM-linear was selected as the predictive model. For Model 2, all eight ML algorithms were evaluated using the same MCCV procedure with only age, CK18, and MGB as features. Notably, SVM-linear Model 2 slightly outperformed Model 1 (Table 3). Based on performance and cost considerations, SVM-linear using age, CK18, and MGB was ultimately proposed for the predictive model.

**Table 3:** Performances of eight ensemble classifiers

	Model 1 (5 features)		Model 2 (3 features)	
	AUC (95% CI)	CER (SD)	AUC (95% CI)	CER (SD)
SVM (linear)	0.70 (0.59, 0.81)	0.35 (0.04)	0.71 (0.59, 0.82)	0.35 (0.04)
GBM	0.70 (0.59, 0.81)	0.34 (0.05)	0.71 (0.60, 0.82)	0.34 (0.05)
SVM (radial)	0.70 (0.58, 0.81)	0.34 (0.04)	0.70 (0.58, 0.81)	0.34 (0.04)
SVM (polynomial)	0.68 (0.56, 0.79)	0.38 (0.06)	0.69 (0.58, 0.80)	0.39 (0.06)
SVM (sigmoid)	0.66 (0.55, 0.79)	0.35 (0.04)	0.60 (0.48, 0.72)	0.43 (0.06)
RF	0.67 (0.55, 0.79)	0.35 (0.05)	0.68 (0.57, 0.80)	0.35 (0.05)
Adaboosting	0.64 (0.52, 0.77)	0.38 (0.04)	0.64 (0.52, 0.76)	0.40 (0.05)
XGB	0.61 (0.49, 0.73)	0.40 (0.05)	0.57 (0.44, 0.69)	0.41 (0.05)

Model 1: age, CK18, MGB, WBC, and platelet. Model 2: age, CK18, and MGB

*AUC* area under the ROC curve, *CI* confidence intervals, *SD* standard deviation, *CER* classification error rate

### Visualizing predicted probabilities

**Fig. 4:**
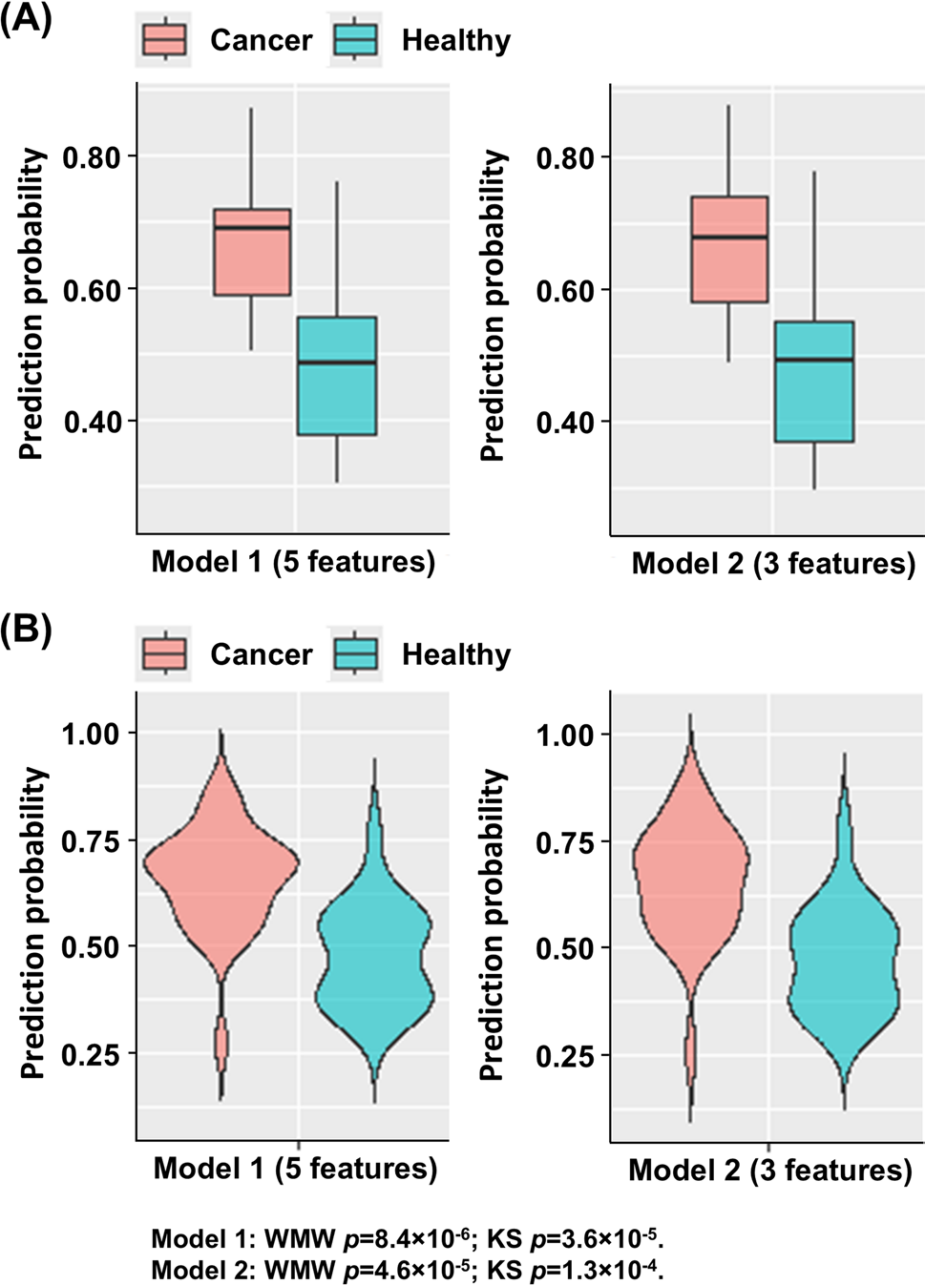
Predicted probabilities generated by Model 1 and Model 2 in the test dataset. **A** Boxplots showing the prediction probabilities of Model 1 (five features: age, CK18, MGB, WBC, and platelet) and Model 2 (three features: age, CK18, and MGB). **B** Violin plots of prediction probabilities from Model 1 and Model 2 (x-axis: groups; y-axis: prediction probability). The significant separation between cancer and benign/healthy groups demonstrates the models’ ability to provide a quantifiable risk score. The width of each violin represents the density of samples at each prediction probability. For each model, the distributions of predicted probabilities for cancer vs. benign/healthy groups were compared using the Wilcoxon–Mann–Whitney (WMW) and Kolmogorov–Smirnov (KS) tests. Model 1: WMW *p* = 8.4 × 10^− 6^; KS *p* = 3.6 × 10^− 5^. Model 2: WMW *p* = 4.6 × 10^− 5^; KS *p* = 1.3 × 10^− 4^

**Fig. 5:**
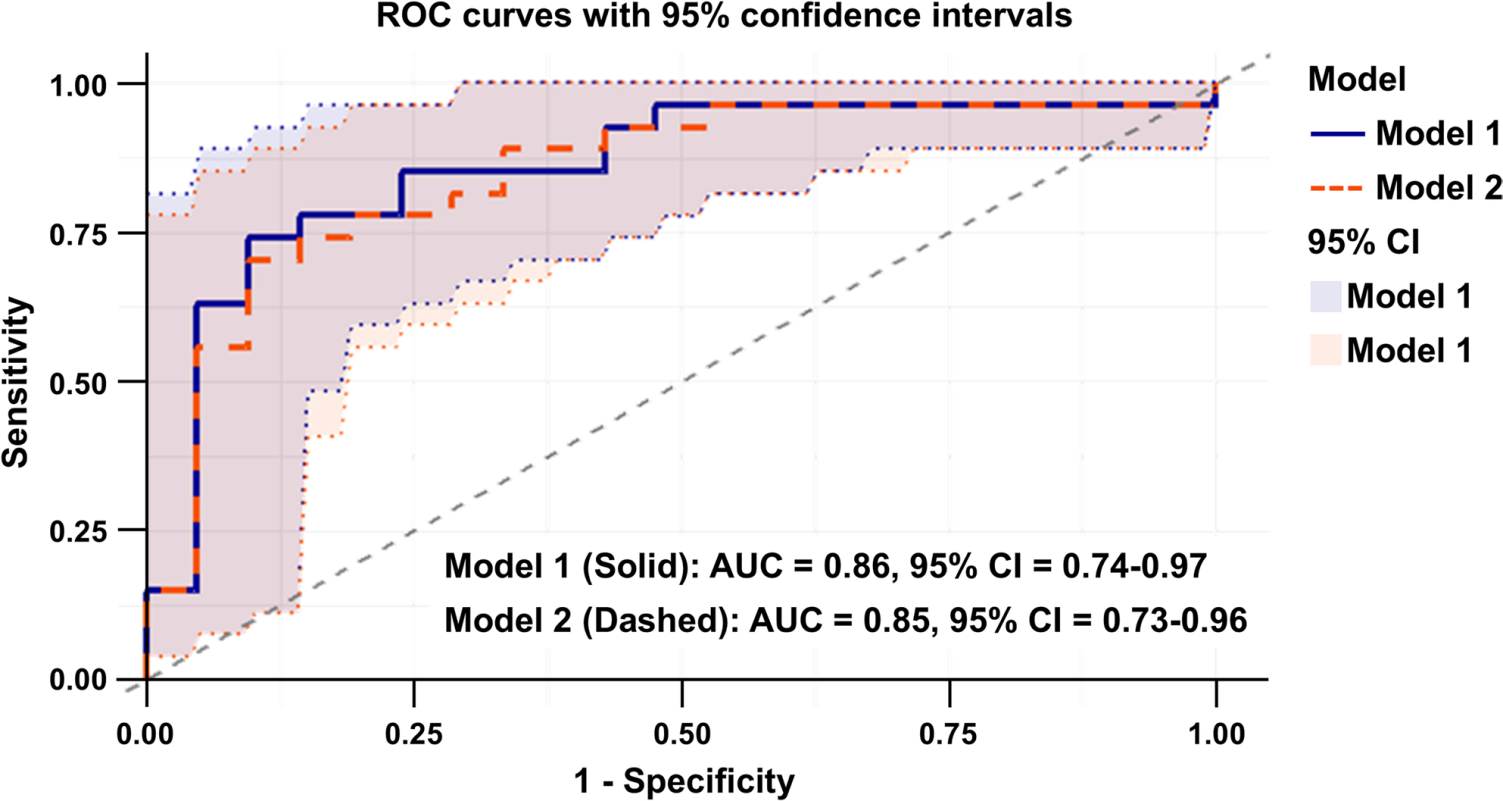
Receiver operating characteristic (ROC) curves of Models 1 and 2 for the test data (*n* = 48). ROC analysis was performed to evaluate the classification performance of the ensemble machine-learning models. Model 1 (solid blue line) included age, CK18, MGB, WBC, and platelet as features, while Model 2 (dashed red line) included age, CK18, and MGB only. The ROC curves for both models demonstrate strong overall diagnostic performance (AUC > 0.85). From a clinical perspective, the steep initial rise of the curves indicates that the models can achieve high sensitivity even at relatively low false-positive rates. The shaded areas indicate 95% CIs generated from 2,000 stratified bootstrap replicates, while CIs for the AUCs were estimated using DeLong’s method

Model performance of the aforementioned SVM-linear classifier was evaluated using an independent test set (*N* = 48, Table S3). Average predicted probabilities stratified by cancer status are summarized (Table 4). In Model 1, the ensemble classifier yielded higher predicted probabilities for cancer patients compared with healthy individuals (mean 0.68 vs. 0.48), achieving high sensitivity (0.96) and NPV (0.92), with moderate specificity (0.52). Similar results were observed in Model 2 (mean 0.66 vs. 0.47), with sensitivity of 0.93 and specificity of 0.57 (Table 4). Sensitivity analysis excluding cases with missing WBC or platelet data (complete-case cohort, *n* = 365) demonstrated preserved sensitivity (0.97) and overall predictive behavior, though specificity decreased (0.22), likely reflecting reduced sample size and estimate instability (Table 5). Performance at the 0.5 threshold was consistent across probability cutoffs from 0.3 to 0.8 (Figure S2), indicating minimal impact of imputation on model characteristics. Predicted probabilities were consistently higher in cancer patients, as shown in boxplots and violin plots (Fig. 4), with significant differences confirmed by Wilcoxon–Mann–Whitney and Kolmogorov–Smirnov tests. ROC analysis demonstrated comparable discrimination for Models 1 and 2, with AUCs of 0.86 and 0.85, respectively (Fig. 5). Exploratory subgroup analyses showed robust performance across cancer stages (accuracy > 90%), molecular subtypes (lowest in ER–/HER2– at 0.80), and mammographic density categories, with reduced accuracy in Category B attributable to a single misclassified case (Tables S5–S7). Overall, the model demonstrated consistent predictive performance across clinicopathological subgroups, supporting its potential utility for malignancy risk characterization.

**Table 4:** Confusion matrices of the ensemble classifiers using SVM-linear based on models 1 and 2 (validation cohort, *n* = 48)

Validation data Model 1 (5 features)			Validation data Model 2 (3 features)		
	True Benign/Healthy	True Cancer		True Benign/Healthy	True Cancer
Predicted Benign/Healthy	11	1	Predicted Benign/Healthy	12	2
Predicted Cancer	10	26	Predicted Cancer	9	25
Predicted probability, mean (±SD)	0.48 (±0.12)	0.68 (±0.12)	Predicted probability Mean (±SD)	0.47 (±0.12)	0.66 (±0.13)
Specificity Sensitivity Accuracy rate PPV NPV	0.52 0.96 0.77 0.72 0.92		Specificity Sensitivity Accuracy rate PPV NPV	0.57 0.93 0.77 0.74 0.86	

Results are based on 1000 votes in the SVM-linear machine learning model. The high sensitivity and NPV suggest that the model is effective in ruling out malignancy, thereby potentially reducing unnecessary follow-up among low-risk individuals

*SD* standard deviation, *WBC* white blood cell count, *PPV* positive predictive value, *NPV* negative predictive value

**Table 5:** Sensitivity analysis of the SVM-linear model using complete-case data

Validation data Model (all covariates) With only complete-case		
	True Benign/Healthy	True Cancer
Predicted Benign/Healthy	4	1
Predicted Cancer	14	29
Predicted probability Mean (±SD)	0.58 (±0.12)	0.68 (±0.11)
Specificity Sensitivity Accuracy rate PPV NPV	0.22 0.97 0.69 0.67 0.80	

*PPV* positive predictive value, *NPV* negative predictive value

### Complementary performance of ML-bases CTC algorithm with breast imaging BIRAD 3/4 category

As an exploratory analysis, we evaluated the model’s performance within the BI-RADS 3 and 4 subgroups, categories that often present diagnostic challenges [31, 32]. These categories comprise the major population of clinical interest and may benefit from additional complementary assessments. A total of 394 patients underwent either breast ultrasound or mammography for screening or diagnostic purposes. For each individual, the highest BI-RADS score from either modality was used for analysis (Table 2). All cases classified as BI-RADS 5 or 6 were malignant. When BI-RADS 3 and 4 patients were combined (*n* = 215), the overall cancer detection rate was 57.7%. Since the ML models were trained on a dataset of 350 patients, performance evaluation was conducted exclusively in the testing cohort (*n* = 48). Among these, 24 individuals were categorized as BI-RADS 3 or 4, with 15 pathologically confirmed as breast cancer (62.5%). In this subgroup, both Models 1 and 2 correctly identified all 15 cancer cases (sensitivity = 1.00). Model 2 showed a modest improvement in specificity (0.44 vs. 0.33) and accuracy (0.79 vs. 0.75) compared with Model 1 (Table 6). Given the limited size of this subgroup, these findings are exploratory and intended to demonstrate proof-of-concept for complementary risk assessment rather than clinical triage.

**Table 6:** Confusion matrices and diagnostic performance of ML-based CTC models stratified by BI-RADS 3 and 4 categories in the testing cohort (*n* = 24)

Model	Characteristic	True Benign/Healthy	True Cancer	Sensitivity	Specificity	PPV	NPV	Accuracy
Model 1 (5 features)	Predicted Benign/Healthy	3	0	1.00	0.33	0.71	1.00	0.75
	Predicted Cancer	6	15					
Model 2 (3 features)	Predicted Benign/Healthy	4	0	1.00	0.44	0.75	1.00	0.79
	Predicted Cancer	5	15					

Model 1: age, CK18, MGB, WBC, and platelet. Model 2: age, CK18, and MGB

*BI-RADS* Breast Imaging Reporting and Data System, *PPV* positive predictive value, *NPV* negative predictive value

## Discussion

In this study, we developed a ML-based model to optimize age, peripheral cell counts, and biomarker data from the detection of circulating tumor cells, aiming to enhance breast cancer risk assessment. This study serves as a proof-of-concept rather than a head-to-head diagnostic comparison. The conventional implementation of ML is to develop a classifier based on randomly sampled training and validation datasets. However, this sampling method may be sensitive to the sampled data, particularly when dealing with moderate or smaller sample sizes, resulting in high variation in model performance [15, 33]. To overcome this problem, we proposed a novel ML strategy using MCCV to integrate multiple classifiers within a random forest structure, thereby reducing heterogeneity due to sampling. Eight ML cores were evaluated accordingly, and the ensemble using SVM with a linear kernel was selected. While this MCCV-based ensemble strategy aims to reduce sampling heterogeneity, it does not eliminate the risk of overfitting, particularly given the modest size of the independent test cohort (*n* = 48). Consequently, the reported performance metrics should be viewed as preliminary estimates, and further external validation remains indispensable.

Recent studies suggest that platelets may enhance CTC survival in the bloodstream and promote cancer metastasis [34]. In the present study, peripheral platelet and leukocyte counts were examined as potential factors influencing CTC enumeration. However, the influence of these parameters was minimized in the final model. The exclusion of WBC is consistent with previous studies using the same CTC detection platform [35], in which automated image analysis excludes cells with leukocyte characteristics. For CTC-based biomarkers, MGB expression has been shown highly variable and substantially lower in TNBC than in luminal-type tumors [36], which may partly explain reduced model performance observed in the ER–/HER2– subgroup (Table S6). Moreover, our findings reflect the biological heterogeneity of TNBC, underscoring the need to evaluate complementary mesenchymal or immune-related markers [36–38]. To ensure more robust performance across molecular subtypes, future model calibration will prioritize feature expansion, including the incorporation of additional non-EpCAM markers such as vimentin [39, 40], to better detect tumors with low MGB expression. In addition, variability observed across BI-RADS breast density categories indicates that breast density may influence CTC detectability or marker expression (Table S7). Collectively, these subgroup analyses provide important guidance for biomarker refinement and suggest that future model calibration incorporating subgroup-aware feature expansion, such as molecular subtype and BI-RADS density as model inputs, may also improve robustness, stabilize feature contributions, and enhance generalizability across heterogeneous patient populations.

Reported PPVs for BI-RADS 3/4 lesions vary widely across studies due to differences in imaging technology, classification standards, and population characteristics [41, 42]. Previous literature reports malignancy risks for the suspicious subcategories at approximately 13% for 4 A and 36% for 4B [41], whereas studies from specialized centers report higher PPVs for BI-RADS 4B and 4 C (approximately 75% and 83%, respectively) [42]. This spectrum indicates the diagnostic ambiguity and heterogeneity of malignancy risk within intermediate BI-RADS categories. Within this imaging-defined context, our Model 2 achieved a PPV of 75% in the BI-RADS 3/4 test subgroup. Importantly, this exploratory finding is descriptive and should not be interpreted as a direct head-to-head diagnostic comparison with BI-RADS subclassifications or as evidence of equivalence to imaging-derived malignancy risk estimates. Rather than replacing imaging, the model may provide complementary information to help characterize risk within BI-RADS 3/4 cases.

The traditional serum tumor marker CA 15 − 3 is used to monitor therapy in advanced breast cancer. However, it has not been validated for the early detection of breast cancer due to a lack of sensitivity and specificity in identifying early-stage breast cancer [43]. Reported sensitivities for combinations of serum tumor markers remain below 60% in recurrent or metastatic disease [44]. In this study, model 2 achieved an NPV of 0.86 and a PPV of 0.74, suggesting its potential to support diagnostic decision-making and reduce unnecessary follow-up for low-risk individuals. While its sensitivity remained high (0.93) with a primary specificity of 0.57, our findings indicate that such performance estimates may also depend on data completeness (Tables 4 and 5). This vulnerability was illustrated by a sensitivity analysis using Model 1, where specificity declined to 0.22 upon restricting the cohort to complete cases (Table 5). This discrepancy highlights the inherent instability of estimates in small datasets and highlights how missing measurements can alter cohort composition. Consequently, these findings must be interpreted strictly as a proof-of-concept. The current false-positive rate remains a significant limitation; beyond the burden of follow-up imaging, it can induce patient anxiety and increase healthcare costs, potentially offsetting the economic benefits of the assay. To mitigate the high false-positive rate, future work will focus on threshold optimization and exploring combined imaging approaches to enhance specificity. Within this context, the use of decision-curve analysis will be essential to identify the optimal net benefit and minimize the clinical and emotional impact of false-positives. While our results provide a proof-of-concept, any impact on resource utilization or clinical workflow remains speculative. Translation into routine practice will require formal health-economic modeling and evaluation of practical factors, such as laboratory turnaround time, to assess clinical potential.

This study has several limitations. Although all benign and healthy participants were cancer-free at blood collection and remained so during at least two years of follow-up, longer surveillance is required to clarify the longitudinal dynamics and lead time of CTC biomarkers. The EpCAM-based enrichment strategy may underrepresent CTCs undergoing epithelial–mesenchymal transition or with low EpCAM expression [36–38]. Patients with LCIS were excluded because these lesions are frequently radiologically occult and typically detected incidentally at biopsy [19, 45], which may introduce selection bias and warrants future dedicated investigation. In addition, the modest size of the independent validation cohort and restriction to Taiwanese women limit generalizability. Accordingly, the performance metrics reported here should not be assumed to directly generalize to other ethnicities or screening populations, where baseline risk, breast density, and screening pathways differ, particularly as Asian women, including Taiwanese populations, tend to have denser breast tissue and an earlier onset of breast cancer compared with Western cohorts [46, 47]. Collectively, larger, multi-center, and multi-ethnic prospective external cohorts will be required to confirm robustness. Furthermore, future studies must focus on evaluating critical translational factors such as physician acceptance and clinical transparency. To address these requirements, developing model interpretability through feature importance or coefficient estimates to clarify the individual contributions of age, CK18, and MGB will be essential after larger external validation. Such analyses will help enhance clinical trust and ensure the model’s successful integration into clinical diagnostic workflows.

## Conclusions

Our study demonstrates the feasibility of combining CTC enumeration with ML for breast cancer detection. Further studies are required to determine whether this approach can provide clinically meaningful complementarity to conventional imaging. While the CTC-based model shows promising discriminative performance, its modest specificity and the relatively small test cohort warrant cautious interpretation. Therefore, its potential role as a complementary risk assessment tool must be thoroughly validated in larger, independent cohorts before any consideration for incorporation into clinical diagnostic workflows.

## Supplementary Information

Supplementary Material 1.

Supplementary Material 2.

## Data Availability

All data generated or analyzed during this study are included in this published article and its supplementary information files.
