# Supplementary material for: Circulating tumor cells (CTCs) enumeration and machine-learning based diagnostic biomarkers for breast cancer detection
Source: BMC Cancer. 2026 Mar 3;26:448. doi: 10.1186/s12885-026-15741-9 (PMC13063714; doi:10.1186/s12885-026-15741-9)
Supplement: Supplementary file 1 — Supplementary Material 1. [file 12885_2026_15741_MOESM1_ESM.docx]

**Supporting information**

## Table S1. The considered hyperparameters for the various models conducted by R software

| Machine learning algorithms | hyperparameters |
| --- | --- |
| SVM (linear) | cost=0.01, 0.1 ,1 ,10 ,100 ,1000 |
| GBM | interaction.depth = c(1, 3, 5, 7, 9)  shrinkage = c(0.01, 0.1, 0.2)  n.minobsinnode = c(5, 10, 20)  bag.fraction =c(0.5, 0.7, 1)  n.trees = 10000 |
| SVM (radial) | cost=0.01, 0.1 ,1 ,10 ,100 ,1000  gamma =0.001, 0.2, 0.5 ,1 ,2 ,3 ,4 |
| SVM (polynomial) | cost=0.1, 1 ,10  gamma =0.001, 1  degree=2, 3, 4, 5  coef0=0.001, 0.1, 1 |
| SVM (sigmoid) | cost=0.1, 1 ,10  gamma =0.001, 0.2, 0.5 ,1  coef0= 0.001, 0.01, 0.1, 1, 10 |
| RF | Tune by the function *tune.rfsrc* |
| Adaboosting | minsplit.par= 10, 20, 30, 40, 50  cp.par=0.001, 0.01, 0.1  maxdepth.par=1, 2, 3, 4, 5, 6, 10, 20 |
| XGB | eta = 0.01, 0.1, 0.2  max_depth = 1, 3, 5, 7, 9  min_child_weight = 1, 3, 5, 7, 9  subsample = 0.7, 0.8, 1.0  colsample_bytree = 0.7, 0.8, 1.0 |

## Table S2. Demographic characteristics of training data.

| Characteristics | All patients (n=350) | Cancer (n=201) | Benign/Healthy (n=149) | *p*-value |
| --- | --- | --- | --- | --- |
| Age (years) | 51 (43, 62) | 55 (46, 63) | 47 (35, 55) | <0.001* |
| CK18 | 2 (1, 6) | 3 (1, 7) | 2 (1, 4) | <0.001* |
| MGB | 4 (2, 9) | 5 (2, 10) | 3 (1, 7) | <0.001* |
| WBC (/uL) | 6400 (5400, 7700) | 6600 (5500, 7700) | 6300 (5400, 7700)^§^ | 0.431 |
| Platelet (/uL) | 256500 (217000, 293000) | 254000 (217000, 289000) | 260000 (215000, 297000)^§^ | 0.518 |
| ^§^Summarized by deleting the missing values (n=28 in Benign/Healthy group)  Median (Q1, Q3)  Mann–Whitney–Wilcoxon test. *p-values < 0.05 were considered statistically significant for comparisons between cancer and benign/healthy groups.  CK18, cytokeratin 18; MGB, mammaglobin; WBC, white blood cell. | | | | |

## Table S3. Demographic characteristics of test data.

| Characteristics | All patients (n=48) | Cancer (n=27) | Benign/Healthy (n=21) | *p*-value |
| --- | --- | --- | --- | --- |
| Age (years) | 51.5 (42.75, 63) | 58 (51, 67.5) | 42 (33, 51) | <0.001* |
| CK18 | 3 (1, 6.25) | 3 (2, 7) | 2 (1, 4) | 0.138 |
| MGB | 4 (2, 7.25) | 4 (2, 8.5) | 4 (2, 7) | 0.484 |
| WBC (/uL) | 6300 (5400, 7000) | 6000 (5300, 6600) | 6500 (5900, 7150)^§^ | 0.242 |
| Platelet (/uL) | 242000 (207000, 299000) | 242000 (203500, 299000) | 245500 (218000, 293250)^§^ | 0.802 |
| ^§^Summarized by deleting the missing values (n=5 in Benign/Healthy group)  Median (Q1, Q3)  Mann–Whitney–Wilcoxon test. *p-values < 0.05 were considered statistically significant for comparisons between cancer and benign/healthy groups.  CK18, cytokeratin 18; MGB, mammaglobin; WBC, white blood cell. | | | | |

## Table S4. Clinical and biological characteristics of patients in the training and testing datasets.

| Characteristics | All patients (n=398) | Training (n=350) | Test (n=48) | *p*-value |
| --- | --- | --- | --- | --- |
| Age (years) | 51 (43, 62) | 51 (43, 62) | 51.5 (42.75, 63) | 0.861 |
| CK18 | 3 (1, 6) | 2 (1, 6) | 3 (1, 6.25) | 0.555 |
| MGB | 4 (2, 8) | 4 (2, 9) | 4 (2, 7.25) | 0.642 |
| WBC (/uL) | 6300 (5400, 7700) | 6400 (5400, 7700) | 6300 (5400, 7000) | 0.366 |
| Platelet (/uL) | 256000 (215000, 294000) | 256500 (217000, 293000) | 242000 (207000, 299000) | 0.598 |
| Median (Q1, Q3)  Mann–Whitney–Wilcoxon test.  CK18, cytokeratin 18; MGB, mammaglobin; WBC, white blood cell. | | | | |

## Table S5. Model performance stratified by disease stage.

| Stage | Disease | Non-Disease | Accuracy | Mean_prob | SD_prob |
| --- | --- | --- | --- | --- | --- |
| Healthy | 10 | 11 | 0.52 | 0.48 | 0.12 |
| Stage 0 | 1 | 0 | 1.00 | 0.68 | – |
| Stage 1 | 8 | 0 | 1.00 | 0.67 | 0.10 |
| Stage 2 | 10 | 1 | 0.91 | 0.64 | 0.17 |
| Stage 3 | 6 | 0 | 1.00 | 0.65 | 0.08 |
| Stage 4 | 1 | 0 | 1.00 | 0.71 | – |

Accuracy, the accuracy rate of the subgroup; Mean_prob, mean predicted probability; SD_prob, standard deviation of predicted probability.

## Table S6. Model performance stratified by breast cancer subtypes.

| Subtypes | Disease | Non-Disease | Accuracy | Mean_prob | SD_prob |
| --- | --- | --- | --- | --- | --- |
| Healthy | 10 | 11 | 0.52 | 0.48 | 0.12 |
| ER+HER2− | 18 | 0 | 1.00 | 0.67 | 0.11 |
| ER+HER2+ | 1 | 0 | 1.00 | 0.56 | – |
| ER−HER2+ | 3 | 0 | 1.00 | 0.69 | 0.004 |
| ER−HER2− | 4 | 1 | 0.80 | 0.61 | 0.19 |

ER, estrogen receptor; HER2, human epidermal growth factor receptor 2; Accuracy, the accuracy rate of the subgroup; Mean_prob, mean predicted probability; SD_prob, standard deviation of predicted probability.

## Table S7. Model performance stratified by mammographic breast density.

| Breast density | Characteristic | Predicted Cancer | Predicted Benign/Healthy | Accuracy | Mean_prob | SD_prob |
| --- | --- | --- | --- | --- | --- | --- |
| Category B | True Cancer | 1 | 0 | 0.50 | 0.72 | 0.21 |
|  | True Benign/Healthy | 1 | 0 |  |  |  |
| Category C | True Cancer | 23 | 1 | 0.71 | 0.61 | 0.13 |
|  | True Benign/Healthy | 9 | 2 |  |  |  |
| Not available | True Cancer | 2 | 0 | 1.00 | 0.45 | 0.13 |
|  | True Benign/Healthy | 0 | 9 |  |  |  |

Accuracy, the accuracy rate of the subgroup; Mean_prob, mean predicted probability; SD_prob, standard deviation of predicted probability.

Category B: scattered areas of fibroglandular density, Category C: heterogeneously dense.


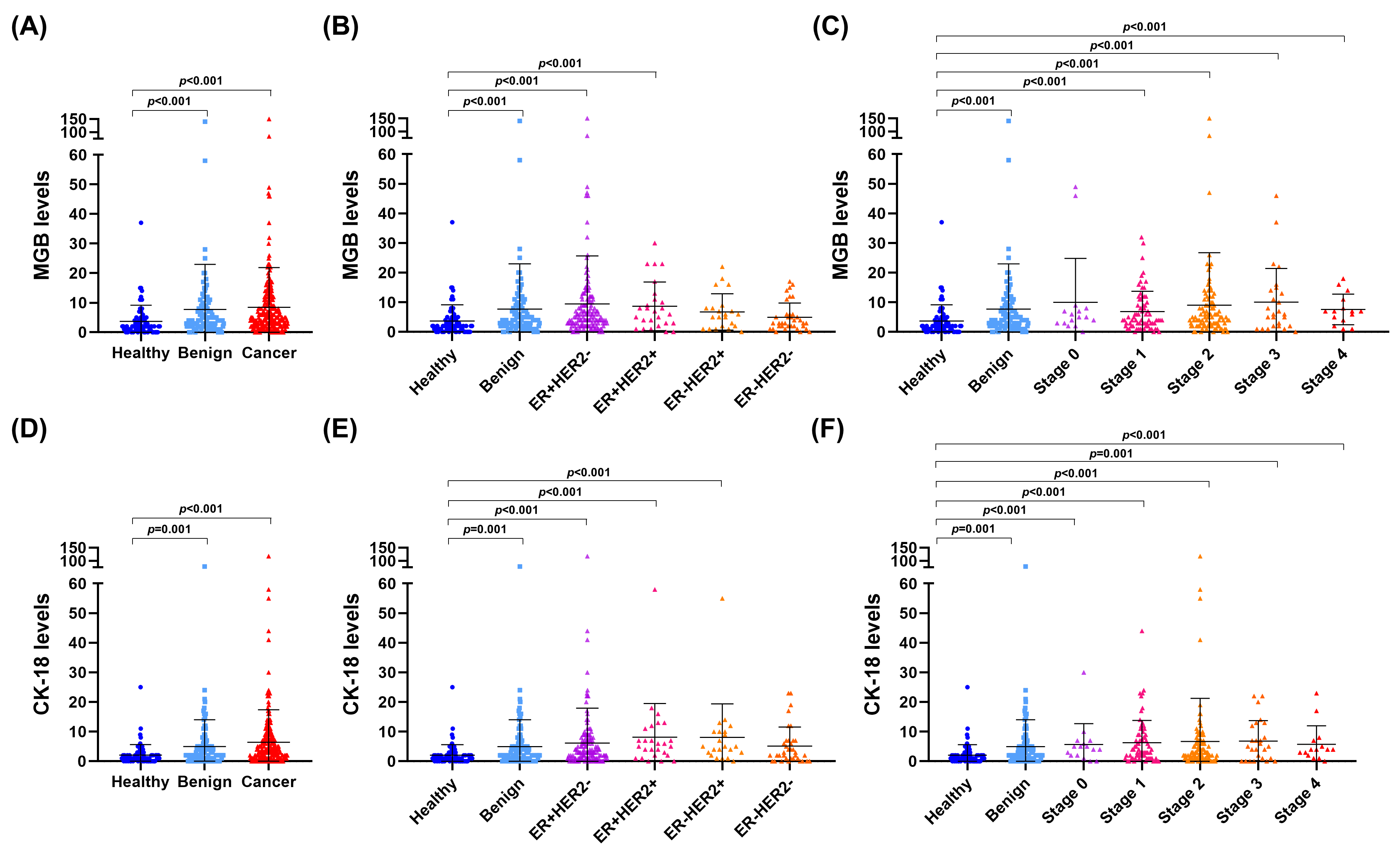


**Figure S1. Expression profiles of CK-18 and MGB in circulating tumor cells (CTCs).**

(A–C) Distributions of MGB expression in CTCs across sample types (A), breast cancer subtypes (B), and cancer stages (C). (D–F) Distributions of CK-18 expression in CTCs across sample types (D), breast cancer subtypes (E), and cancer stages (F). The Kruskal–Wallis test was used to evaluate differences among groups. When significant differences were detected, post hoc pairwise subgroup comparisons were performed. To correct for multiple testing, Bonferroni adjustment was applied by dividing the original alpha value by the number of comparisons. Post hoc tests were conducted for multiple comparisons in each panel, with the number of tests and adjusted significance thresholds as follows: panels A and D, 3 tests (*p* < 0.017); panels B and E, 15 tests (*p* < 0.003); and panels C and F, 21 tests (*p* < 0.002).


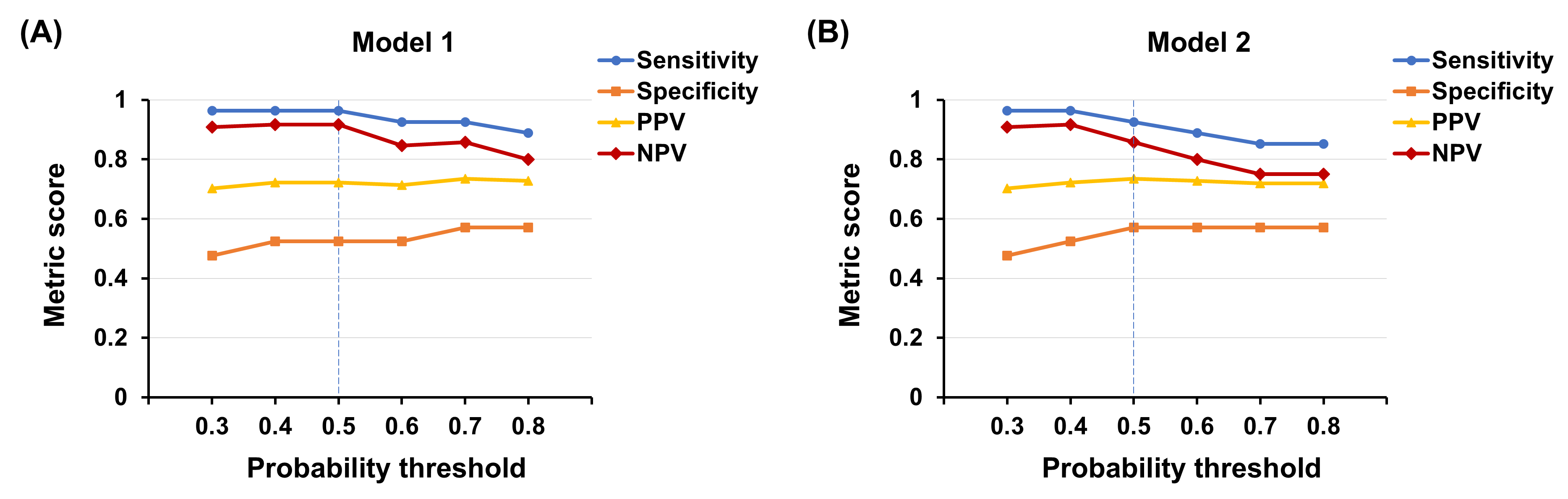


**Figure S2. Model performance across different probability thresholds.**

Performance metrics, including sensitivity (blue), specificity (orange), positive predictive value (PPV, yellow), and negative predictive value (NPV, dark red), are evaluated across probability thresholds ranging from 0.3 to 0.8 for (A) Model 1 and (B) Model 2. The vertical dashed line indicates the pre-specified threshold of 0.5. The stability of these metrics across a broad range of thresholds underscores the robustness of the ensemble classifier. From a clinical decision-making perspective, the high sensitivity and NPV maintained at lower thresholds (0.3–0.5) highlight the model's primary utility as a reliable rule-out tool for malignancy. Conversely, the gradual increase in specificity at higher thresholds demonstrates the potential for threshold optimization to minimize false-positive burden and associated patient anxiety, depending on the specific clinical requirements for risk stratification.
