# Supplementary material for: Circulating tumor cells (CTCs) enumeration and machine-learning based diagnostic biomarkers for breast cancer detection
Source: BMC Cancer. 2026 Mar 3;26:448. doi: 10.1186/s12885-026-15741-9 (PMC13063714; doi:10.1186/s12885-026-15741-9)
Supplement: Supplementary file 2 — Supplementary Material 2. [file 12885_2026_15741_MOESM2_ESM.docx]

# **Title:**

**Circulating tumor cells (CTCs) enumeration and machine-learning based diagnostic biomarkers for breast cancer detection**

**Authors**

Chun-Yu Liu^1,2,3^, Yu-Hsiang Lin^1^, Yi-Fang Tsai^1,2,4^, Po-Yen Lu^5^, Ji-Lin Chen^1,2^, Yu-Hsuan Li^5^, Chi-Cheng Huang^2,4,6^, Yen-Shu Lin^1,2,4^, Ta-Chung Chao^1,2,3^, Chin-Jung Feng^1,2,4^, Chih-Yi Hsu^1,7^, Jen-Hwey Chiu^2,4,8^, Chyong-Mei Chen^5,*^, Ling-Ming Tseng^1,2,4,*^

**Affiliations**

1School of Medicine, College of Medicine, National Yang Ming Chiao Tung University, Taipei 11217, Taiwan.

2Comprehensive Breast Health Center, Department of Surgery, Taipei Veterans General Hospital, Taipei 11217, Taiwan.

3Division of Medical Oncology, Department of Oncology, Taipei Veterans General Hospital, Taipei 11217, Taiwan.

4Division of Breast Surgery, Department of Surgery, Taipei Veterans General Hospital, Taipei 11217, Taiwan.

5Institute of Public Health, College of Medicine, National Yang Ming Chiao Tung University, Taipei, Taiwan.

6Department of Public Health, College of Public Health, National Taiwan University, Taipei, Taiwan.

7Department of Pathology and Laboratory Medicine, Taipei Veterans General Hospital, Taipei 11217, Taiwan.

8Institute of Traditional Medicine, School of Medicine, National Yang Ming Chiao Tung University, Taipei 11217, Taiwan.

| This folder contains the R code for implementing the proposed method. This program can be run with R version 4.2.2 or later. The program requires four packages—“e1071,” “missForest,” “pROC,” and “ROCR”—all of which are automatically installed by the provided program. The input dataset was "read.data.csv", which is not currently available for public release due to data ownership and restrictions imposed by the hospital. The data that support the findings of this study are available from the corresponding author, upon reasonable request.  To use the new code, please conduct the following two steps.  Step 1: Implement the code “code_SVM_linear_Model.r” to construct the svm-forest machine. In this file, readers need to determine the features by setting biomarker.name=c("Age", "CK18", "MGB", "WBC","Platelet") or biomarker.name=c("Age", "CK18", "MGB").  Step 2: Implement “code_prediction for test data.r” to predict the test data, test_data.csv. |
| --- |

# **Details**

The provided code package includes two scripts: **code_SVM_linear_Model.r** and **code_prediction_for_test_data.r**.

- **code_SVM_linear_Model.r** is used to construct the ensemble model and generates two output files: svm_bestmodel.rds and imp.train.X_1000.rds.
- **code_prediction_for_test_data.r** is used to evaluate the performance of the ensemble model on the independent test dataset.

The following provides a step-by-step walkthrough of the proposed method, demonstrating how the ensemble model is trained and subsequently applied for prediction.

**code_SVM_linear_Model.r**

1. **Feature selection**
   Users first specify the features by setting, for example,

| # R  biomarker.name = c("Age", "CK18", "MGB", "WBC", "Platelet") |
| --- |

or

| # R  biomarker.name = c("Age", "CK18", "MGB") |
| --- |

1. **Data import**
   The dataset is imported using

| # R  read.data = read.csv("read.data.csv", fileEncoding = "big5") |
| --- |

In our analysis, the dataset contained a total of 398 subjects. The input data "read.data.csv" contain variables, ID, Age, CK18, MGB, WBC and Platelet. The data were split into two datasets as training data and test data, respectively.

1. **Model training**
   - The required R packages are installed automatically.
   - Sampling was performed according to the number of cancer cases and non-cancer/healthy individuals to ensure that both the training and test datasets preserved the same structure as the original data.
   - The test dataset consisted of 48 subjects, as described in the main article.
   - The remaining 350 subjects were repeatedly partitioned using a Monte Carlo cross-validation (MCCV) scheme. In each iteration, the data were randomly split into a training set (75%, *n*=262) and a validation set (25%, *n*=88).
   - Within each split, machine learning models were trained on the training data using 10-fold cross-validation.
   - A support vector machine (SVM) with a linear kernel was trained for each split.
   - This procedure was repeated 1000 times, and the resulting “small” models were aggregated to form the ensemble classifier.

**code_prediction_for_test_data.r**

- - The best-performing ensemble predictive model was used to generate predictions for the test data, based on 1,000 constituent learners of the selected model. Each individual learner classified a patient as having breast cancer if the predicted probability exceeded the clinical threshold of 0.5.
  - Final classifications were produced using a majority-vote aggregation of predictions from the 1,000 constituent learners of the selected model.

This script applies the ensemble classifier to the test dataset. The output includes the predicted probabilities, predicted disease status, and performance metrics such as the ROC curve and AUC.

# **sessionInfo:**

R version 4.2.2 (2022-10-31 ucrt)

Platform: x86_64-w64-mingw32/x64 (64-bit)

Running under: Windows 10 x64 (build 26200)

Matrix products: default

locale:

[1] LC_COLLATE=Chinese (Traditional)_Taiwan.utf8 LC_CTYPE=Chinese (Traditional)_Taiwan.utf8 LC_MONETARY=Chinese (Traditional)_Taiwan.utf8

[4] LC_NUMERIC=C LC_TIME=Chinese (Traditional)_Taiwan.utf8

attached base packages:

[1] stats graphics grDevices utils datasets methods base

loaded via a namespace (and not attached):

[1] dplyr_1.1.4 grid_4.2.2 R6_2.6.1 lifecycle_1.0.4 gtable_0.3.6 magrittr_2.0.3 scales_1.4.0

[8] ggplot2_3.5.1 pillar_1.11.0 rlang_1.1.3 cli_3.6.2 farver_2.1.1 vctrs_0.6.5 generics_0.1.4

[15] RColorBrewer_1.1-3 glue_1.7.0 compiler_4.2.2 pkgconfig_2.0.3 tidyselect_1.2.1 tibble_3.2.1

# **Appendix 1: R Script for SVM Linear Model Construction**

code_SVM_linear_Model.r

########################################

# construct the svm-forest machine #

########################################

rm(list=ls())

# Determine the features by setting biomarker.name

biomarker.name=c("Age", "CK18", "MGB", "WBC","Platelet")

#############################################################################################

list.of.packages = c("e1071", "missForest", "pROC", "ROCR")

new.packages = list.of.packages[!(list.of.packages %in% installed.packages()[,"Package"])]

if(length(new.packages)) install.packages(new.packages, repos='http://cran.us.r-project.org')

library(missForest)

library(e1071)

library(pROC)

library(ROCR)

mrep=1000

bestmod_list=list()

svm.linear=matrix(NA, mrep, 5)

colnames(svm.linear)=c("ii", "auc", "CI.L", "CI.U", "error.rate")

read.data=read.csv("read.data.csv",fileEncoding='big5')

ID=c(1:398)

read.data=cbind(read.data,ID)

X.p=length(biomarker.name)

input.data=read.data[, c("Name", biomarker.name, "Sample.Type","ID")]

y=as.factor((read.data[, "Sample.Type"]=="Cancer")*1) # 1: Cancer; 0: Benign/Healthy

original.data=cbind(input.data, y)

healthy=which(original.data[,'Sample.Type']=="Benign" |original.data[,'Sample.Type']=='Healthy')

Cancer=which(original.data[,'Sample.Type']=="Cancer" )

a=table(original.data[,'Sample.Type'])

Healthy_proportion=(a[1]+a[3])/sum(a)

Cancer_proportion=(a[2])/sum(a)

set.seed(100)

seed.mrep=100

mm=48 # size of validation data

test_size_1=round(mm*Healthy_proportion,0)

test_size_2=mm-test_size_1

Healthy_test=sample(healthy, test_size_1)

Cancer_test=sample(Cancer, test_size_2)

test.data=rbind(original.data[Healthy_test,], original.data[Cancer_test,])

write.table(test.data, file="test_data.csv", sep=",", row.names=F, na = "NA", fileEncoding='big5')

test.ID=test.data[,'ID']

svm_data=original.data[-test.ID,]

N.total=dim(original.data)[1]

n=N.total-mm

train.size=round(n*3/4, 0)

imp.train.X_1000=array(NA, dim=c(mrep, train.size, X.p))

dimnames(imp.train.X_1000)=list(rep('SVM.linear', mrep), c(1:train.size), biomarker.name)

ii=1

for (ii in 1: mrep) {

cat("ii=", ii, "\n")

seed.mrep=seed.mrep+1

set.seed(seed.mrep)

train_index = sample(nrow(svm_data), train.size)

train.data = svm_data[train_index, ]

validation.data = svm_data[-train_index, ]

train.X=train.data[, biomarker.name]

train.y=as.factor(train.data[, "y"])

validation.X=validation.data[, biomarker.name]

validation.y=as.factor(validation.data[, "y"])

##################################################################################################################

# Imput missing data. When there is no missing data, the imputation approach will not change the input dataset. #

##################################################################################################################

# 1) impute train

imp.train.X = missForest(train.X)$ximp

imp.train.data=cbind(imp.train.X, train.y)

imp.train.X_1000[ii,,]=as.matrix(imp.train.X)

train.validation.X = rbind(validation.X, imp.train.X)

imp.validation.X = missForest(train.validation.X)$ximp[1:nrow(validation.X), ]

imp.validation.data=cbind(imp.validation.X, validation.y)

#######################################

# Build model SVM with linear kernel #

#######################################

tune.out=tune.svm(train.y~., data = imp.train.data,kernel='linear',

range=list(cost=c(0.01, 0.1 ,1 ,10 ,100 ,1000)),

tunecontrol = tune.control(sampling = "cross", cross=10),

probability=TRUE)

tune.out.linear=tune.out

bestmod=tune.out.linear$best.model

bestmod_list[[ii]]=tune.out.linear$best.model

###########################################

# Calculate ROC & AUC for validation data #

###########################################

pred.table=table(true=validation.data[,"y"], pred=predict(tune.out.linear$best.model, imp.validation.data[, 1:X.p], probability=TRUE, decision.values =TRUE))

pred.validationdata=predict(tune.out.linear$best.model, imp.validation.data, probability=TRUE, decision.values =TRUE, fitted=TRUE)

error.rate.linear=(sum(pred.table)-sum(diag(pred.table)))/dim(imp.validation.data)[1]

cat("svm.error.rate based on linear kernel=", error.rate.linear, "\n")

print(pred.table)

pred_validation = attributes(predict(tune.out.linear$best.model, imp.validation.data, probability=TRUE, decision.values =TRUE, fitted=TRUE))$prob

roc.results=roc(validation.y, pred_validation[, "1"], ci=TRUE)

roc.out=cbind(roc.results$sensitivities, roc.results$specificities, roc.results$thresholds)

colnames(roc.out)=c("sensitivity", "specificity", "threshold")

plot(1-roc.out[,"specificity"], roc.out[,"sensitivity"], type="n", xlab="FPR", ylab="TPR", xlim=c(0,1), ylim=c(0,1),

main="Validation data by SVM with linear kernel \n with imputation")

lines(1-roc.out[,"specificity"], roc.out[,"sensitivity"], lwd=2)

abline(0, 1, lty=2)

text(0.7, 0.3, paste("AUC=", round(roc.results$auc, 2)))

text(0.7, 0.2, paste("95 % CI =", round(roc.results$ci[1],2), "-", round(roc.results$ci[3],2)))

svm.linear[ii, ]=c(ii, roc.results$auc, as.numeric(roc.results$ci)[1], as.numeric(roc.results$ci)[3], error.rate.linear)

}

cat("linear:", "\n"); print(round(apply(svm.linear, 2, mean), 2)[-1])

cat("linear(sd):", "\n"); print(round(apply(svm.linear, 2, sd), 2)[-1])

write.table(svm.linear, "svm.linear(model1).txt", quote=F)

saveRDS(bestmod_list, file = "svm_bestmodel.rds", ascii = FALSE, version = NULL, compress = TRUE, refhook = NULL)

saveRDS(imp.train.X_1000, file="imp.train.X_1000.rds", ascii = FALSE, version = NULL, compress = TRUE, refhook = NULL)

# **Appendix 2: R Script for Test Data Prediction**

code_prediction for test data.r

##################################

# svm_forest for test data #

##################################

rm(list=ls())

list.of.packages = c("e1071", "missForest", "pROC", "ROCR")

new.packages = list.of.packages[!(list.of.packages %in% installed.packages()[,"Package"])]

if(length(new.packages)) install.packages(new.packages, repos='http://cran.us.r-project.org')

library(missForest)

library(e1071)

library(pROC)

library(ROCR)

bestmod_list=readRDS(file = "svm_bestmodel.rds")

imp.train.X_1000=readRDS(file = "imp.train.X_1000.rds")

test.data=read.csv("test_data.csv",fileEncoding='big5')

mrep=length(bestmod_list)

mm=nrow(test.data)

biomarker.name=c("Age", "CK18", "MGB", "WBC","Platelet")

pred_test.out=array(NA, dim=c(mrep, mm, 3))

voting.out=matrix(NA, mm, 2)

probability=matrix(NA,mm, 1)

colnames(voting.out)=c("Voting.y", "True.y")

forest_data.X=test.data[, biomarker.name]

forest_data.y=test.data[, "y"]

for (i in 1:mrep) {

forest.train.X = rbind(forest_data.X, imp.train.X_1000[i,,])

imp.forest.X = missForest(forest.train.X)$ximp[1:nrow(forest_data.X), ]

imp.forest_data=cbind(imp.forest.X, forest_data.y)

out= attributes(predict(bestmod_list[[i]], imp.forest_data, probability=TRUE, decision.values =TRUE, fitted=TRUE))$prob

pred_test.out[i, ,]=cbind(rep(i, mm), out[,'1'], (out[,'1']>.5)*1)

}

for (k in 1:mm) {

voting.out[k,]=c((apply(pred_test.out[,k,], 2, mean)[3]>=0.5)*1 , as.numeric(imp.forest_data[k, "forest_data.y"])-1)

probability[k,]=apply(pred_test.out[,k,],2,mean)[2]

}

table.pred=table(voting.out[,1], voting.out[,2])

TP=table.pred[2,2]

FP=table.pred[2,1]

FN=table.pred[1,2]

precision=TP/(TP+FP)

recall=TP/(TP+FN)

specificity=mean(1-voting.out[voting.out[, "True.y"]==0, "Voting.y"])

F1_score=(2*precision*recall)/(precision+recall)

cat("Votint results=", "\n")

print(table.pred)

cat("precision=", precision, "\n")

cat("Sensitivity=", recall, "\n")

cat("Specificity=", specificity, "\n")

cat("F1_score=", F1_score, "\n")

cat("accuracy rate=", sum(diag(table(voting.out[,1], voting.out[,2])))/mm, "\n")

cat("Forest of svm predicted probability=", "\n"); print(apply(pred_test.out[,,2], 2, mean))

new.data=cbind(test.data[, "y"], voting.out,probability)

colnames(new.data)=c('Stage',"Prediction",'True','Probability')

########################

# Draw the ROC curve #

########################

roc.results=roc(new.data[, "True"], new.data[, "Probability"], ci=TRUE)

roc.out=cbind(roc.results$sensitivities, roc.results$specificities, roc.results$thresholds)

colnames(roc.out)=c("sensitivity", "specificity", "threshold")

plot(1-roc.out[,"specificity"], roc.out[,"sensitivity"], type="n", xlab="FPR", ylab="TPR", xlim=c(0,1), ylim=c(0,1),

main="Validation data by SVM with linear kernel \n with imputation")

lines(1-roc.out[,"specificity"], roc.out[,"sensitivity"], lwd=2)

abline(0, 1, lty=2)

text(0.7, 0.3, paste("AUC=", round(roc.results$auc, 2)))

text(0.7, 0.2, paste("95 % CI =", round(roc.results$ci[1],2), "-", round(roc.results$ci[3],2)))

sink("sessionInfo.txt")

sessionInfo()

sink()
